# Sandwich Therapy with Radionuclide Combined with Intralesional Injections Based on Differences in the Spatial Structure Components of Keloids

**DOI:** 10.1007/s00266-024-04442-y

**Published:** 2024-10-14

**Authors:** Xiaolan Ou, Lei Chen, Chunjing Yu, Tao Yan, Biao Huang, Jing Wang, Shuyu Zhang, Guozhong Lyu, Daojiang Yu

**Affiliations:** 1 Department of Plastic and Burn Surgery, The Second Affiliated Hospital of Chengdu Medical College, Nuclear Industry 416 Hospital. No.4, North Section 4, Second Ring Road, Chengdu District, Chengdu, 610051 Sichuan China; 2 Department of Nuclear Medicine, Affiliated Hospital of Jiangnan University, No. 1000, Hefeng Road, Xuelang Street, Binhu DistrictJiangsu province, Wuxi, 214122 China; 3 Sichuan Clinical Medical Research Center of Radiation and Therapy, Nuclear Industry 416 Hospital. No.4, North Section 4, Second Ring Road, Chengdu District, Chengdu, 610051 Sichuan China; 4 The Department of Plastic and Burn surgery, Affiliated Hospital of Jiangnan University, 1000 Hefeng Road, Binhu District, Wuxi, 214122 Jiangsu China

**Keywords:** Keloid, Spatial structure components, Radionuclide, Sandwich therapy, Precision treatment

## Abstract

**Background:**

Complications such as ulceration, depigmentation, and recurrence limit the use of intralesional injections and brachytherapy in keloid treatment. We designed a modified "sandwich" therapy based on the spatial distribution of keloid components to reduce the incidence of these complications.

**Methods:**

First, we analyzed the spatial distribution pattern of scar tissue through single-cell sequencing analysis, ultrasound, and pathology. Subsequently, a "sandwich" therapy combining radionuclide and intralesional injections was designed based on the pattern found in the previous stage. Finally, 40 patients with keloid scars at 41 sites were included in the clinical trial.

**Results:**

Single-cell sequencing identified two significant cellularly highly expressed genes and enriched pathways in the keloid vascular wall that primarily play essential roles in angiogenesis and promoting collagen synthesis, thereby promoting scar growth. Color ultrasound showed that there were hierarchical differences in the blood supply of the keloid, and further H&E, CD34, and eNOS staining showed that there were hierarchical differences in the spatial structure of blood vessels, fibroblasts, and collagen in the keloid. In clinical studies, the complication rate of “sandwich” therapy is lower than that of conventional treatment.

**Conclusion:**

The distribution of blood vessels and collagen in keloid scars is characterized by spatial variability. The "sandwich" therapy of radionuclide combined with intralesional injections is a modified type of precisely targeted therapy designed based on this variability; it has fewer complications and good clinical efficacy and is worthy of popularization.

**Level of Evidence II:**

This journal requires that authors assign a level of evidence to each article. For a full description of these Evidence-Based Medicine ratings, please refer to the Table of Contents or the online Instructions to Authors www.springer.com/00266.

## Introduction

Scars cause pain, itching, and other serious effects on the patient's normal work and life. There are numerous treatment options for scarring. Previous studies have shown that radiotherapy is the only treatment effective in preventing keloid formation [1, 2]. At present, there are three main treatment modalities: electron beam therapy, x-ray therapy, and brachytherapy, all of which have achieved good therapeutic effects [3]. Among them, brachytherapy is highly regarded because of its targeted and concentrated irradiation of the target area. At present, the commonly used radiation sources in clinical practice mainly include Strontium-90 (^90^Sr), ^32^P, and ^192^Ir. Among them, ^90^Sr irradiation of scarring has a more precise clinical efficacy and widely used [1]. Its advantages lie in its simple operation, ease of use, affordability. And because of the shallow depth of invasion, high degree of safety, that is, 80% of the equivalent radiation dose only penetrates 2–3 mm below the skin surface and does not damage deep tissues and neighboring organs. However, because its depth of penetration is limited, the efficacy of a single therapy is still not ideal.

In recent years, intralesional injections combined with radionuclides have been effective and widely used in the treatment and prevention of keloids [4–6]. However, there are systemic adverse reactions (menstrual disorders, Cushing's syndrome, opportunistic infections, etc.) and local adverse reactions (local skin atrophy, hypopigmentation, and capillary dilatation, etc.) resulting from overuse or long-term corticosteroid dose accumulation [7]. In clinical practice, intralesional injections are too shallow, often showing drug adjuvant, local ulceration and so on. For thicker scars, repeated irradiation is used to compensate for the lack of radionuclide penetration, and the accumulation of radiation dose leads to complications such as pigment loss, radioactive damage, and even ulceration. These above problems greatly affect patient satisfaction and limit its popularization and application.

To solve the above problems, the group studied the spatial distribution characteristics and patterns of keloid components and found that there were a gradual decrease in blood vessels and a gradual increase in collagen from the superficial to the deep layers, with a hierarchical difference. Based on the results of the study, the "sandwich" program of radionuclide combined with intralesional injections was designed. The treatment plan was matched and precise according to the differences in the spatial structure components of keloids, utilized the memory response of radiotherapy and the synergistic advantages of radiotherapy and chemotherapy to reduce the amount of medication and radiation dose, reduce adverse reactions, and improve the therapeutic efficacy, and promoted the popularization of the use of intralesional injections and proximity nuclide dressings in the treatment of keloids.

## Materials and Methods

### Inclusion Criteria

(1) Patients with a clear diagnosis of keloids. (2) Disease duration of half a year and above and no tendency of spontaneous atrophy. (3) Sign the informed consent form and agree to regular follow-up within 1 year. (4) Keloid protruding less than 3mm from the body surface.

## Exclusion Criteria

(1) Patients treated for keloids within the past 6 months. (2) Patients with infections or ulcers in or around the keloid. (3) Patients allergic to TAC or 5-Fu.

## Procedure

### Fundamental Research

Keloid tissue and normal skin tissue at the edge of the keloid were selected from five keloid patients. (All keloid specimens were pathologically examined to confirm the diagnosis.) After excision of the tissue specimens, the samples were digested into single-cell suspensions within 30 minutes. The samples that met the sequencing criteria after detection of cellular activity were loaded into the microfluidic device, and then, the prepared single-cell suspensions were sequentially subjected to PCR amplification and library construction, quality control, down-conversion and clustering, analysis of differentially expressed genes (DEGs), and Kyoto encyclopedia of genes and genomes, (KEGG) enrichment analysis. Ten patients with keloid scars and a history of more than six months were randomly selected. Color Doppler ultrasonography was performed to detect the thickness and blood flow of the scar tissue. The detection of H&E, CD34, and eNOS immunohistochemical was carried out on the scar tissue specimens to analyze their spatial characteristics. On this basis, the "sandwich" therapy of radionuclide combined with intralesional injections was rationally designed.

## Clinical Application Research

A total of 40 patients with keloids at 41 sites were included in this study, and one patient had two sites (Shoulder and elbow) considered as two lesions. Two groups were randomized: one group was treated with this regimen ("Sandwich" therapy), and the other was treated with this regimen and a conventional regimen in a self-controlled study: the keloid was divided into two symmetrical zones along the midline of the keloid, A and B. Zone A was treated with the conventional treatment, zone B was treated with the treatment, and the effects and complication rates of the two treatments were compared.

## The Ratio of Drug

Local anesthetics: 5 ml of 2% lidocaine injection and 5 ml of 0.75% ropivacaine hydrochloride injection were added to 10 ml of 0.9% sodium chloride injection; 0.02 mL of epinephrine hydrochloride was added.

Drug A: Add 2 ml of TAC injection (40 mg/mL) to 4 ml of 2.5% 5-FU injection.

Drug B: Add 0.8 ml of 2.5% 5-FU injection to 4 ml of TAC injection (40 mg/mL).

## Conventional TAC and 5-FU Intralesional Injections Combined with Radionuclide (Conventional Therapy)

**Scheme 1:**
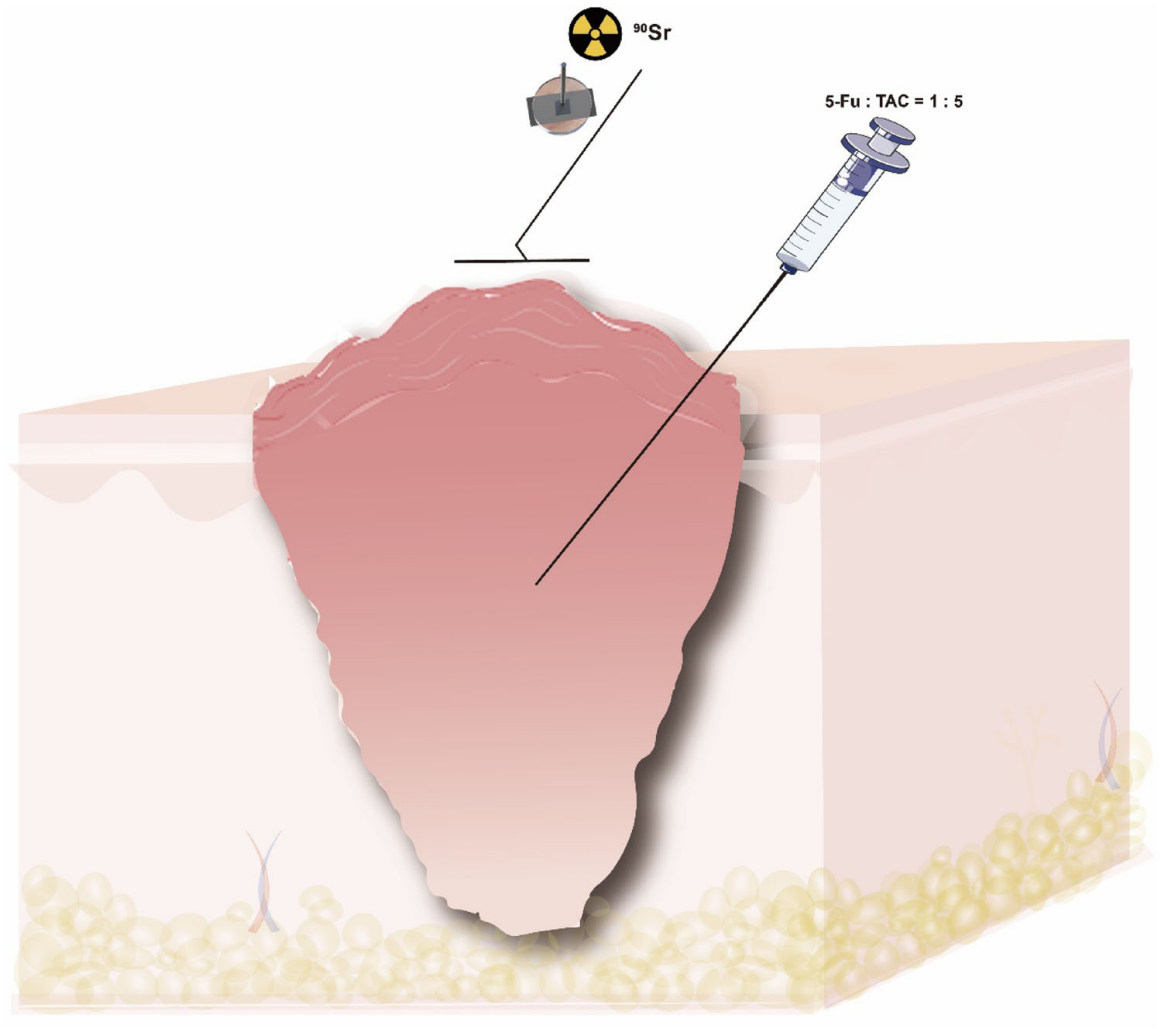
Patterns of traditional treatment methods

Drug B was mixed with 1 ml of local anesthetics; intralesional injections were performed, with a total mixture dose of 0.2 ml/cm^3^; three consecutive injections were performed at 3-week intervals; and 3 weeks after the last injection, the keloid was treated with ^90^Sr brachytherapy after it had become soft or flattened. The keloid lesion area was covered with a 30 × 30 mm ^90^Sr dressing with a 20 × 20 mm effective field (1.81 mGy/s). A lead shield protected the surrounding normal skin. A total dose of 10 Gy, 15 Gy, or 20 Gy was administered for 3 or 4 consecutive days, depending on the area of the body involved. Keloids on the chest wall and the back of the shoulder received a total of 20 Gy of radiation therapy in four sessions over four days; keloids on the earlobe received 10 Gy for three consecutive days; and keloids in other areas received 15 Gy of radiation therapy in three sessions over three days. Dose differences between different body regions are based on different site-specific radiotherapy regimens [8]. Treatment will be discontinued if skin swelling, infection, or ulceration occurs. See Scheme 1 for details.

## "Sandwich" Therapy with Radionuclide Combined with Intralesional Injections (New Therapy)

**Scheme 2:**
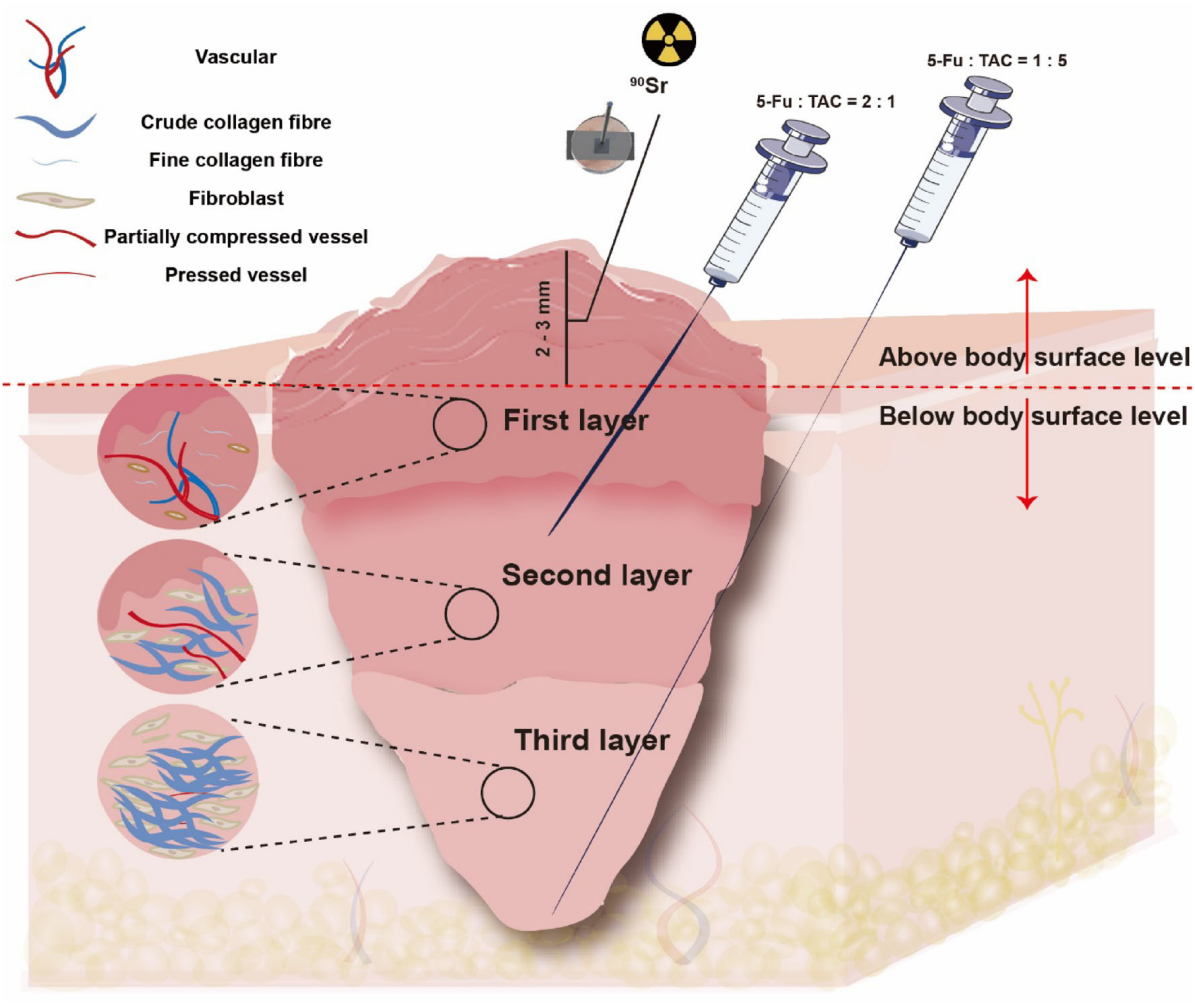
Structural hierarchy of keloid in two planes and three levels and "sandwich" therapy of radionuclide combined with intralesional injections

The keloid was divided into the first, second, and third layers according to the structural differences in the distribution of keloid tissue and its blood vessels. Under ultrasound-guided, drug A was injected intralesionally in the superficial second layer (Drug A was mixed with 1 ml of local anesthetics, and the total mixture dose was 0.2 ml/cm^3^); drug B was injected into the deep part of the third layer (Drug B was mixed with 1 ml of local anesthetics, and the total mixture dose was 0.2 ml/cm^3^). On the next day, ^90^Sr brachytherapy was given to the upper 2.0–3.0 mm of the first layer, and the treatment was carried out once a day for 3–4 consecutive days with the same single dose and number of times as that of the traditional program. The three layers were presented as a “sandwich” type of therapy. See Scheme 2 for details.

## Follow-up Strategy

All patients were followed up for at least 12 months after treatment. Standardized clinical photographs were taken with a digital camera (Canon EOS 100D, Canon Inc, Tokyo, Japan) (with the same camera settings and lighting conditions) before treatment and at 3 and 6 months after treatment; all patients were asked to perform a color Doppler ultrasonography before treatment and at 1 month after treatment to detect and evaluate the lesions. Information on side effects (hyperpigmentation/alopecia, skin ulcers, capillary dilatation, etc.), recurrence, and satisfaction is collected during follow-up.

## Statistical Analysis

Quantitative data are expressed as mean ± standard deviation. Differences in means within groups for quantitative data were tested using paired T-tests, and differences between groups were tested using two independent samples T-tests. Data were statistically analyzed in rows, and graphs were generated using GraphPad Prism version 9 software. *p* < 0.05 was considered statistically different.

## Results

### Fundamental Research

**Fig. 1:**
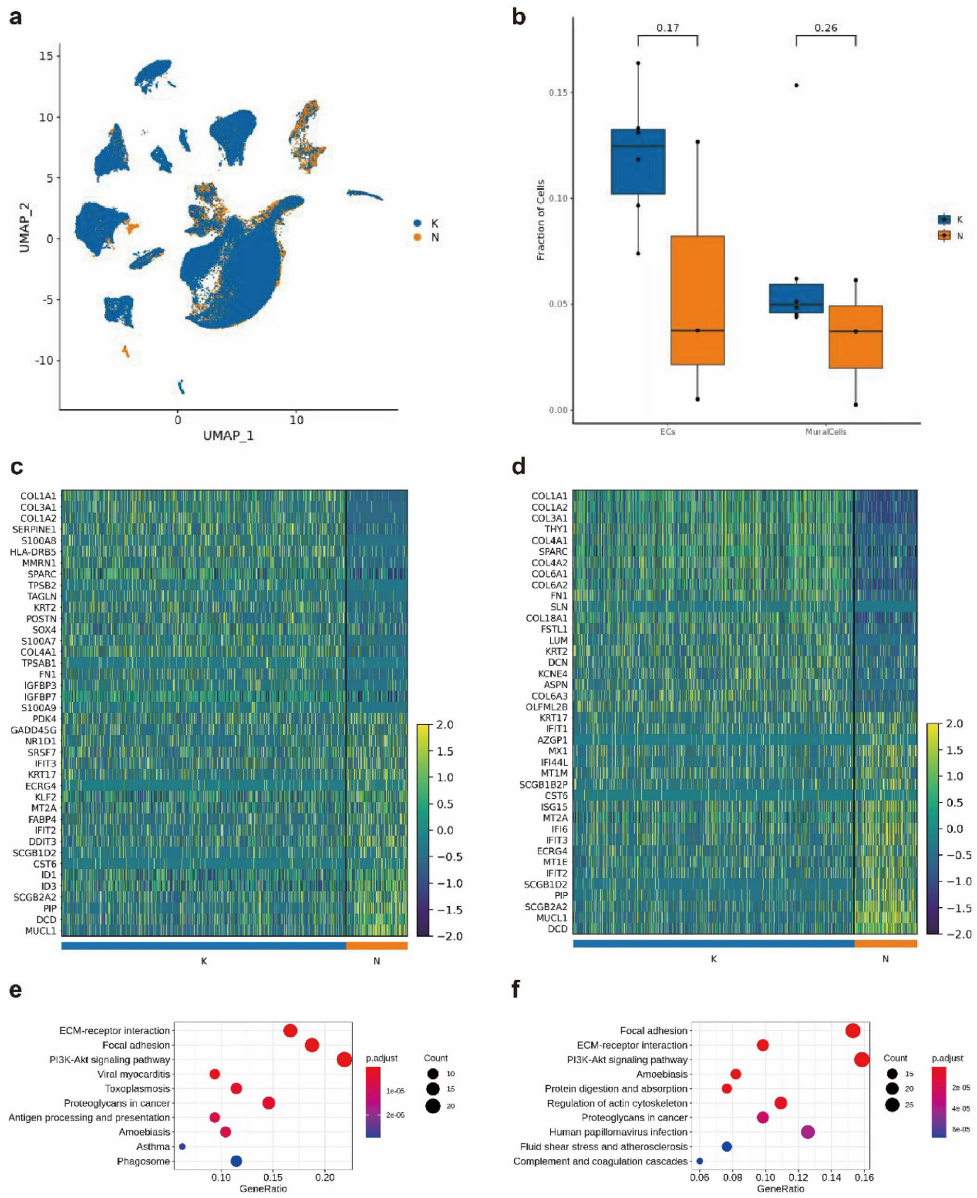
Single-cell sequencing results of keloid and normal skin tissues. **a** UMAP downscaling clustering maps based on keloid and normal skin vasculature classified by origin. **b** Fraction of endothelial and mural cells. **c** Differential gene heatmap of endothelial cells. **d** Differential gene heatmap of mural cells. **e** Endothelial cell differential gene KEGG enrichment analysis. **f** Mural cell differential gene KEGG enrichment analysis. Note: Blue is labeled from scar tissue, and yellow is labeled from normal skin tissue

As shown in Fig. 1a-b, there was a tendency for the vascular wall's primary constituent cells (endothelial cells and mural cells) to increase in keloidal compared to normal skin. Further analysis of the differential genes and functions of endothelial and mural cells revealed that endothelial cells highly expressed genes such as COL1A1, COL3A1, COL1A2, SERPINE1, and S100AB, of which COL1A1, COL3A1, and COL1A2 were associated with collagen synthesis, and SERPINE1 was an inhibitor of fibrinolysis; S100AB was involved in cell cycle progression and differentiation (Fig. 1c); KEGG enrichment analysis revealed that it was associated with classical signaling pathways such as Pl3K-Akt signaling pathway, focal adhesion, and ECM–receptor interaction (Fig. 1e); mural cells highly expressed the COL1A1, COL1A2, COL3A1, THY1, and COL4A1 genes, of which COL1A1, COL1A2, COL3A1, and COL4A1 were related to collagen synthesis; THY1 was involved in cell adhesion and cell communication (Fig. 1d); and KEGG enrichment analysis in classical signaling pathways such as the Pl3K-Akt signaling pathway, focal adhesion, and ECM–receptor interaction (Fig. 1f).

**Fig. 2:**
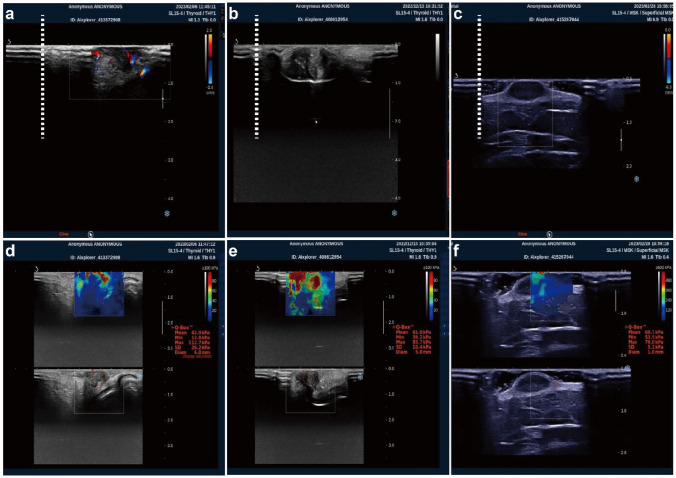
Ultrasound findings of keloid. **a**–**c** Keloid visualization. **d**–**f** Keloid blood flow visualization

As shown in Fig. 2a-c, the whole layer of normal skin consists of two relatively homogeneous and consistent bands of strong echoes sandwiched by a more expansive isoechoic zone in the center. The mean value of the average skin thickness measured was 0.35 ± 0.036 cm, consistent with the normal results. The two echogenic solid bands of the scar were present and continuous, and the echogenicity of the middle layer was not homogeneous. The average thickness of the hyperplastic scar was measured to be 0.4725 ± .025 cm, which was significantly thicker than that of the normal skin, and its thickness depended on the degree of hyperplasia of the scar. As shown in Fig. 2d-f, the blood flow signals in the thickened middle layer of the scar had a high density. Some of them were connected into strips or sheets, which were irregularly arranged, and the blood flow was continuously visualized during dynamic observation. In-depth analysis revealed that there may be structural differences in blood flow in the scar: the first layer has a more prosperous blood supply, which gradually decreases downward.

**Fig. 3:**
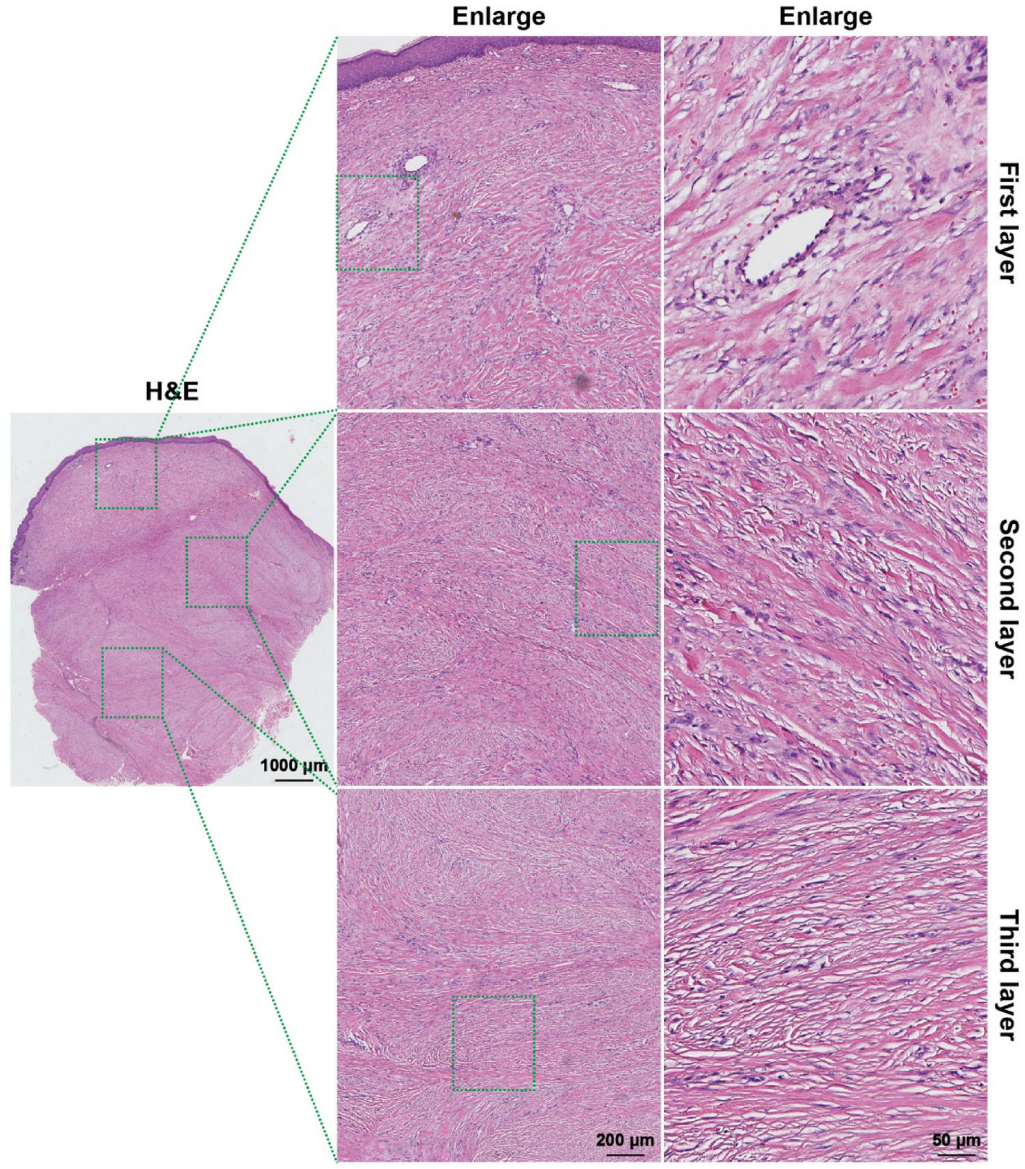
H&E of a keloid. The first layer is mostly immature, fibrillar collagen fibers containing abundant blood vessels and an open official lumen; the second is hypermorphic; and the third is predominantly thick fibrous bundles

**Fig. 4:**
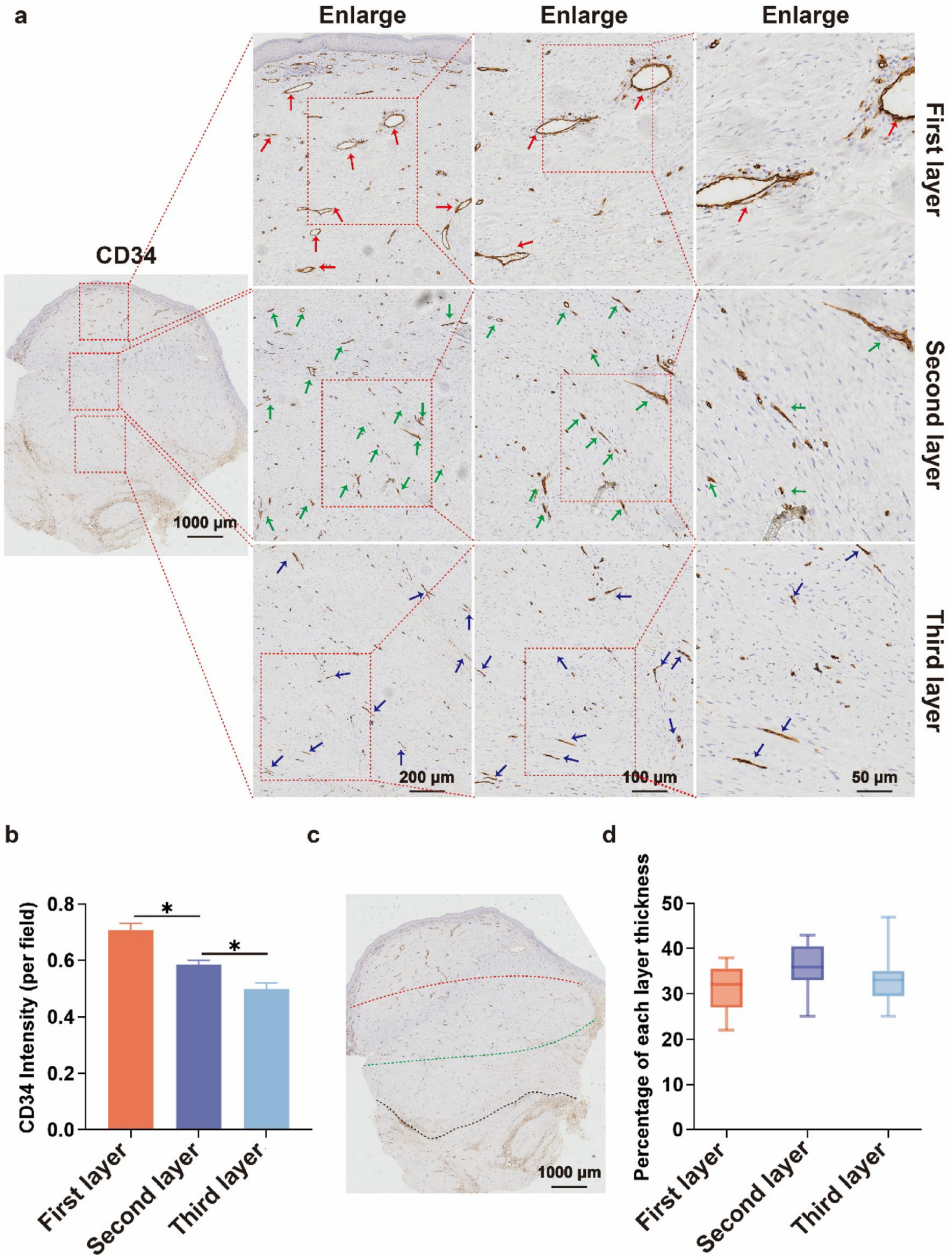
CD34 staining of keloids. **a** CD34 expression in different levels of keloid. **b** Average silver density of CD34 expression in each level of keloid. **c** Stratification of keloid. **d** Percentage (%) of keloid in each level

**Fig. 5:**
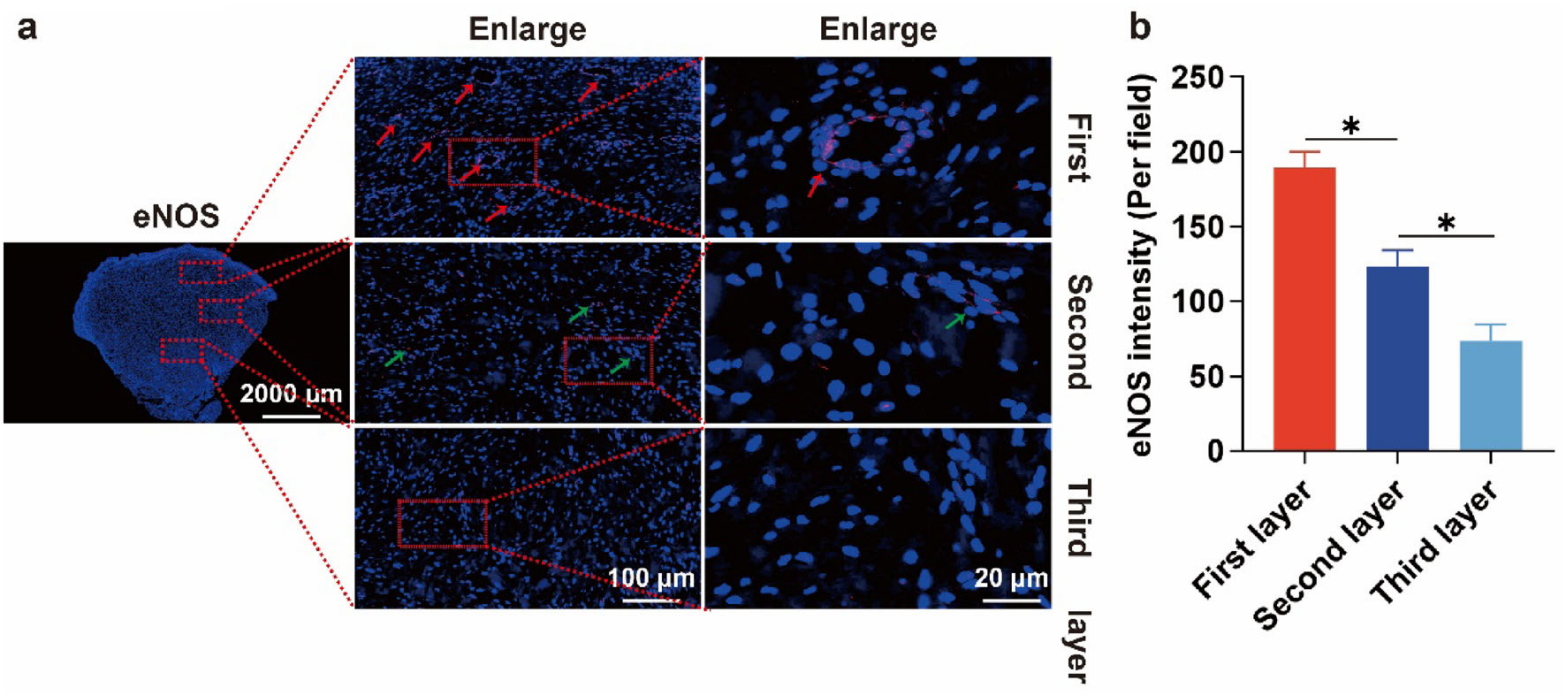
eNOS staining of keloids. **a** eNOS expression in different levels of keloid. **b** Average silver density of eNOS expression in each level of keloid

As shown in Fig. 3, the H&E results of the keloid showed an increase in the number of fibroblasts, an over-accumulation of extracellular matrix (ECM) components (especially collagen), and disorganization of the collagen fibers in the dermis and subcutaneous tissues; this is in line with the findings of previous studies. In-depth analysis revealed structural hierarchical differences in the keloid: the first layer consisted of more immature, more slender collagen fibers with abundant and open blood vessels, which gradually changed downwards; its third layer was mainly dominated by coarse bundles of fibers intertwined with each other to form a network, with no blood vessels seen. Further CD34 immunohistochemical analysis (Fig. 4a) showed that the first layer of the keloid had a large number of blood vessels with open lumens; the number of blood vessels gradually decreased from the first layer downwards (Fig. 4b), and the lumens were gradually closed; the second layer of blood vessels had partially open lumens; the third layer of the keloid had the smallest number of blood vessels with completely closed lumens. Combining the number of vessels and the opening of vascular lumen, the keloid was classified into three layers (Fig. 4c): the first layer, the second layer, and the third layer; further statistical analysis is shown in Fig. 4d: the first layer accounted for 31% ± 6%; the second layer accounted for 36% ± 6%; and the third layer accounted for about 33% ± 6%. Further, eNOS staining (Fig. 5) showed a gradual decrease in the expression of eNOS from top to bottom of the scar, indicating a gradual decline in the function of endothelial cells, which is basically consistent with the above findings. Based on this difference in the structural level of the keloid, the "sandwich" treatment of radionuclide combined with intralesional injections was rationally designed (Scheme 2); it was significantly different from the conventional treatment (Scheme 1). The design of the "sandwich" therapy for keloid scars of different thicknesses is shown in Table 1.

**Table 1:** Therapeutic design of "sandwich" therapy for keloids of different thicknesses

Different thicknesses	Layer	Thickness
> 2 – 4 mm	First layer	2 mm
	Second layer	1 mm
	Third layer	1 mm
> 4 – 5 mm	First layer	2 mm
	Second layer	1.5 mm
	Third layer	1.5 mm
> 5 – 6 mm	First layer	2 mm
	Second layer	2 mm
	Third layer	2 mm
> 6 – 7 mm	First layer	2 mm
	Second layer	2.5 mm
	Third layer	2.5 mm

## Clinical Research

**Fig. 6:**
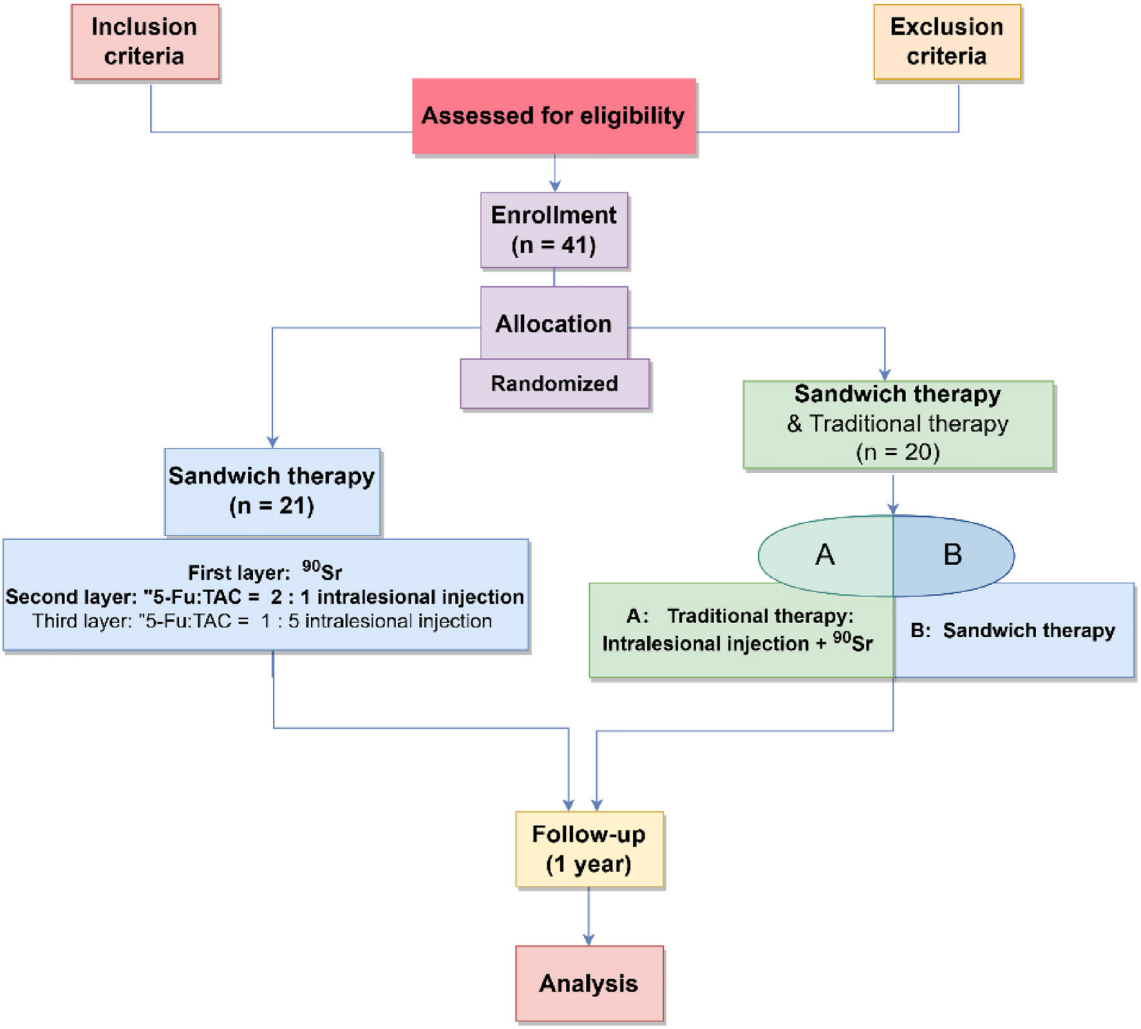
Flow chart of this study

**Fig. 7:**
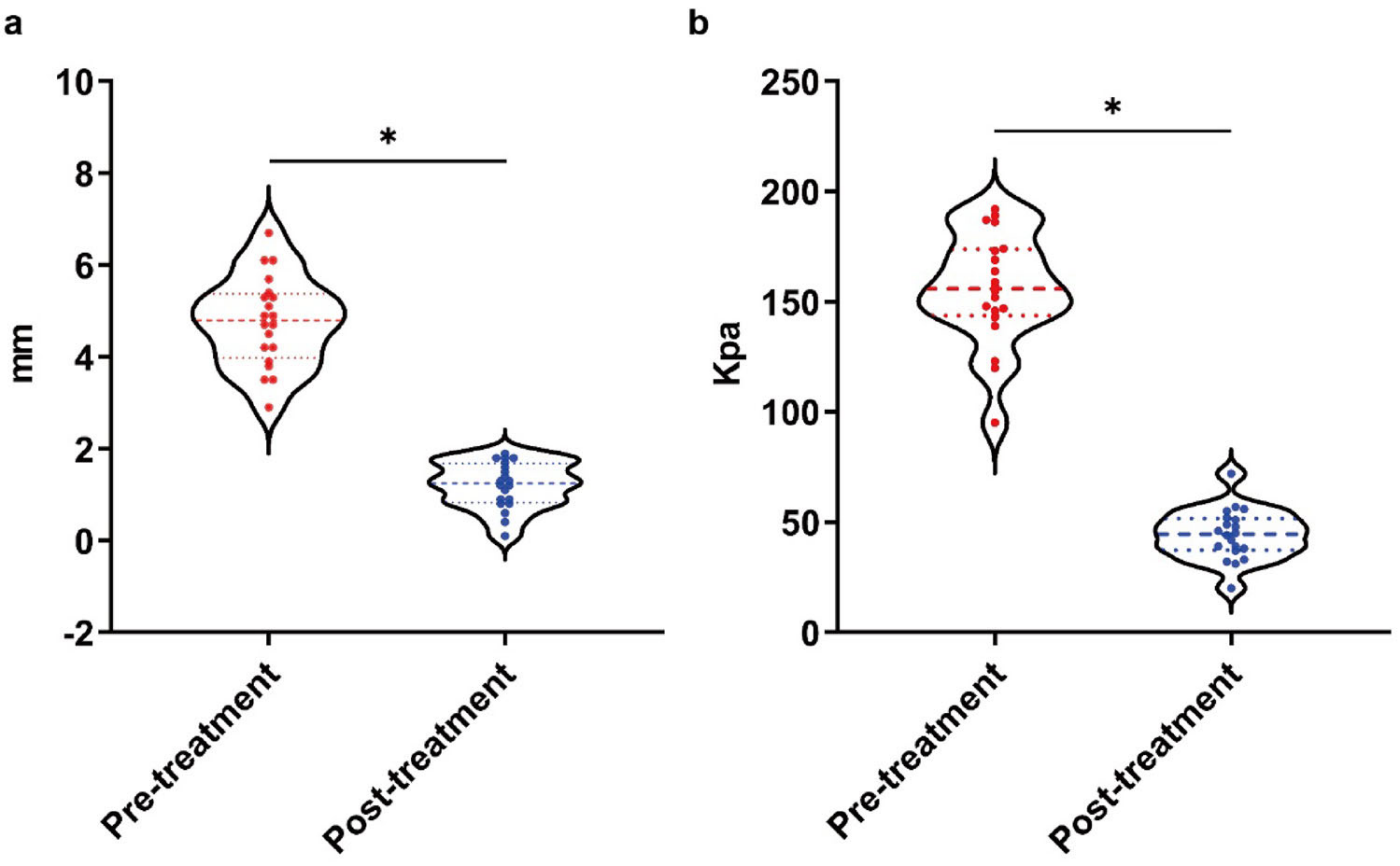
Effect of new therapy on keloid thickness and hardness. The new treatment reduced the thickness (**a**) and hardness (**b**) of the keloid as measured by Doppler ultrasound. *p* < 0.05

**Fig. 8:**
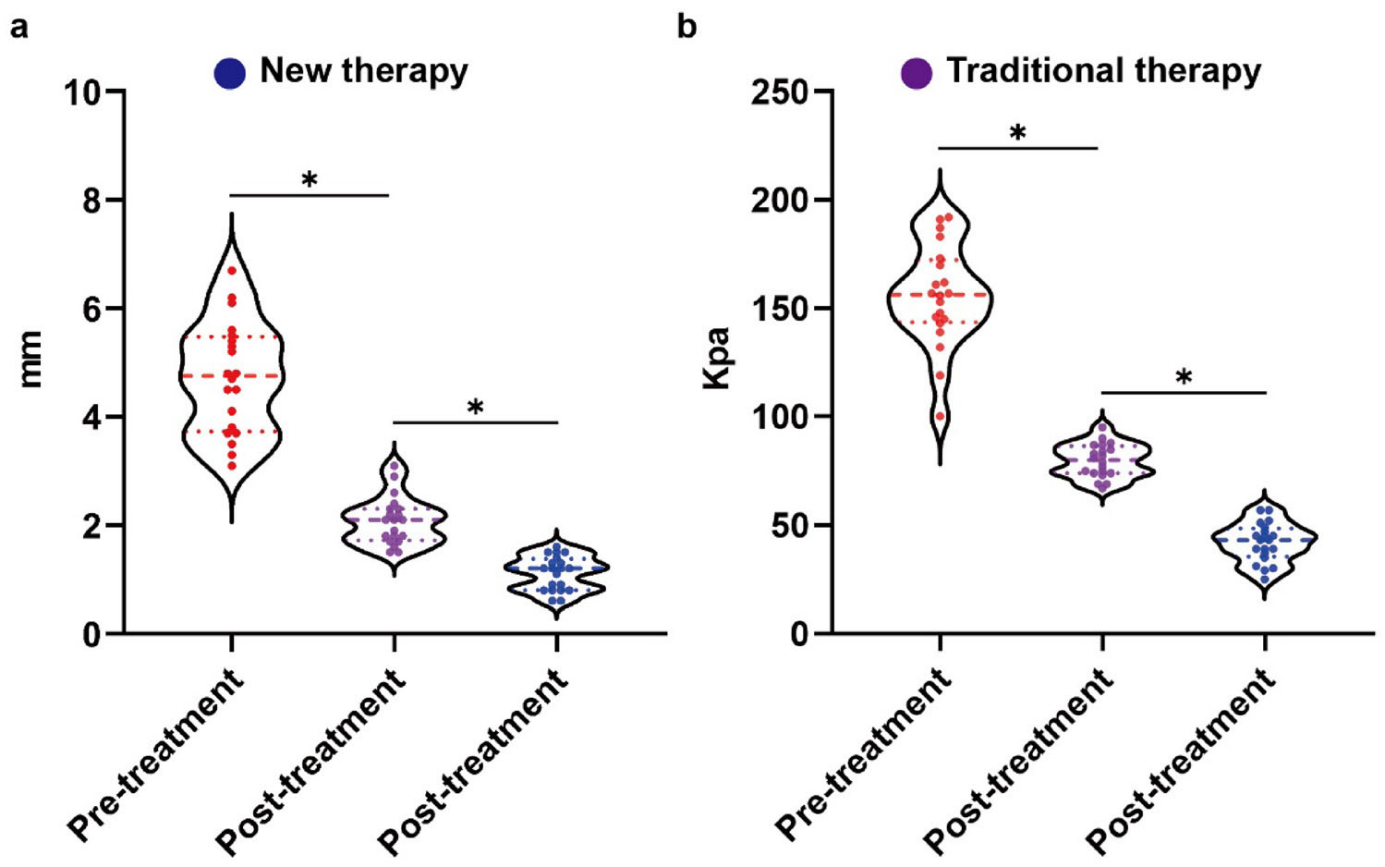
Effect of new versus conventional therapy on keloid thickness and hardness. The new therapy reduced the thickness (**a**) and hardness (**b**) of keloids measured by Doppler ultrasound more significantly than the traditional therapy, *p* < 0.05

We recruited a total of 40 patients with 41 lesions; one patient with two sites (Shoulder and elbow) was considered as two lesions; the lesion sites included the ear, thorax, scapulae, abdomen, and limbs, of which the ear was the most common; the main causes of keloids included surgery, trauma, acne, and vaccination (Table 2). A total of 21 keloids in 20 patients were treated with the "sandwich" therapy of radiotherapy combined with intralesional injections; to exclude individual differences, a total of 20 keloids in another group of 20 patients were treated with this treatment plan and the traditional treatment plan (Fig. 6). Color Doppler ultrasonography was performed before and 1 month after treatment. As shown in Fig. 7, ultrasound imaging of soft tissue thickness and modulus of elasticity, the new treatment was effective in reducing the thickness of keloids and changing their stiffness. The results of further self-controlled experiments showed that the new treatment reduced the thickness and hardness of keloid more significantly than the traditional treatment, as shown in Fig. 8a: the new treatment reduced the thickness of keloid by an average of 3.62 mm (3.62 ± 0.91 mm), while the traditional treatment reduced the thickness of keloid by 2.65 mm (2.65 ± 0.80 mm). As shown in Fig. 8b, the new treatment improved the stiffness of the keloid more significantly than the conventional treatment, which reduced the modulus of elasticity by 113.8 Kpa (113.8 ± 25.46 Kpa), compared to the traditional medicine, which reduced the modulus of elasticity by 76.0 Kpa (76.0 ± 23.26 Kpa). At the 12-month follow-up, the new treatment resulted in one recurrence (4.8%) and one localized ulceration (4.8%), with a patient satisfaction rate of 90.5%. In contrast, 10% of patients with conventional therapy had a partial recurrence, 5% had a partial recurrence, and 10% had local ulceration; moreover, 4 patients reported difficulty adhering to multiple treatments during treatment.

**Table 2:** Statistical information on patient recruitment and keloids

	New therapy	New & traditional therapy
Age(years): median	30(21-59)	30(21-59)
Medical history	7.09 ± 5.99	7.20 ± 5.85
Gender	20	20
Female	14 (70%)	14 (70%)
Male	6 (30%)	6 (30%)
Sites	21	20
Ear	7 (33.3%)	6 (28.6%)
Neck and chest	6 (28.6%)	8 (28.6%)
Scapula	2 (9.5%)	2 (9.5%)
Abdomen	2 (9.5%)	2 (9.5%)
Limbs	4 (19.0%)	2 (9.5%)
Cause of keloids		
Surgery	12 (29.3%)	11 (26.8 %)
Trauma	2 (4.9%)	3 (7.3%)
Burn and scald	2 (4.9%)	2 (4.9%)
Acne	1 (2.4%)	1 (2.4%)
Vaccination	4 (9.8%)	3 (7.3%)

**Fig. 9:**
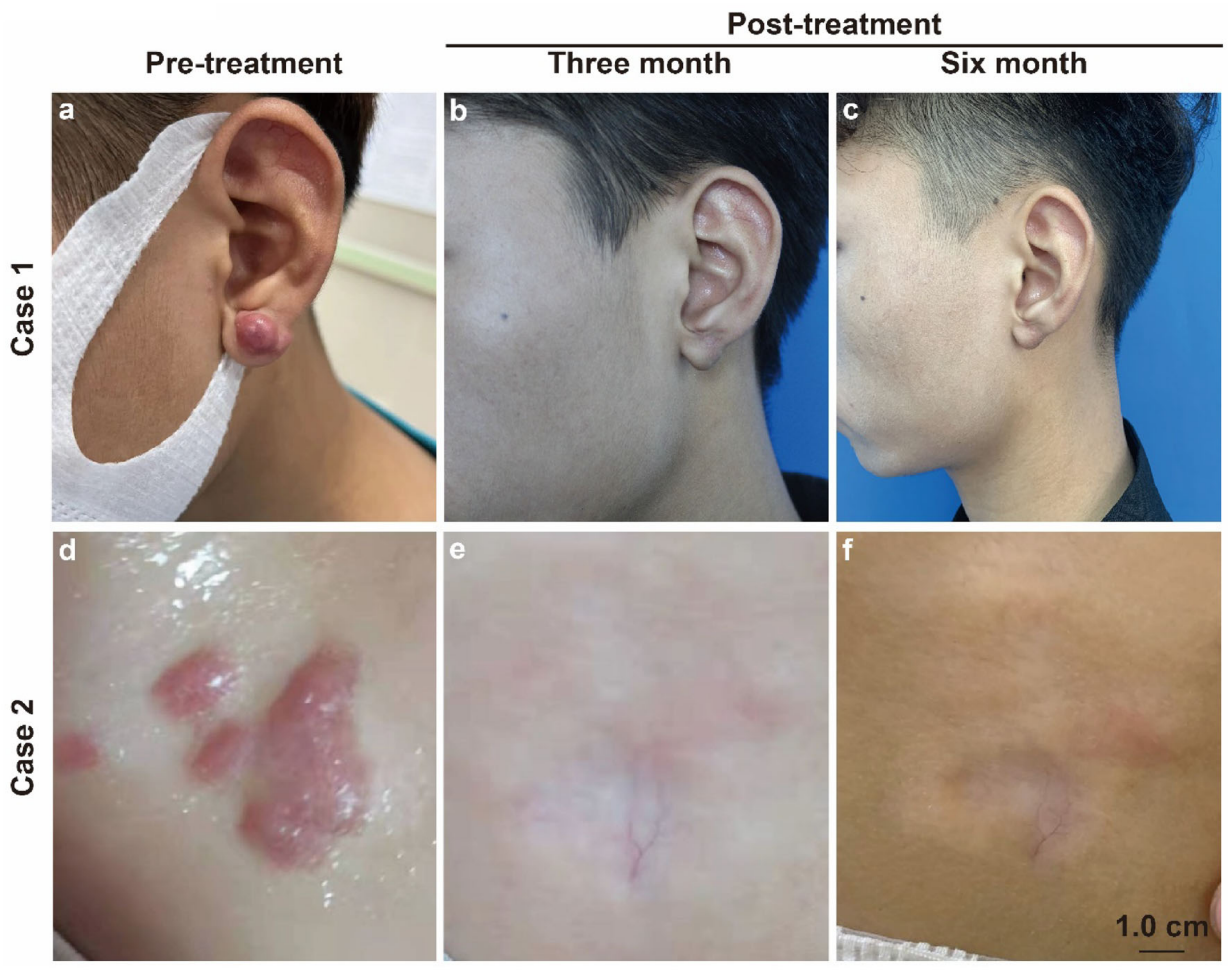
Case 1: a 25-year-old male patient with earlobe keloid; Case 2: a 23-year-old female with keloid in the anterior chest area; changes in the keloid before treatment, 3 months, and 6 months after treatment with the new therapy. Scale bar, 1.0 cm

We report 2 representative cases, as shown in Fig. 9. The appearance of the keloid was significantly improved at 3 months and 6 months after treatment with the new therapy. During the follow-up, the patient's pain and itching symptoms significantly improved, achieving patient satisfaction and no related side effects. It shows that the treatment program designed based on the differences in spatial structural components of a keloid can effectively treat keloid scars.

**Fig. 10:**
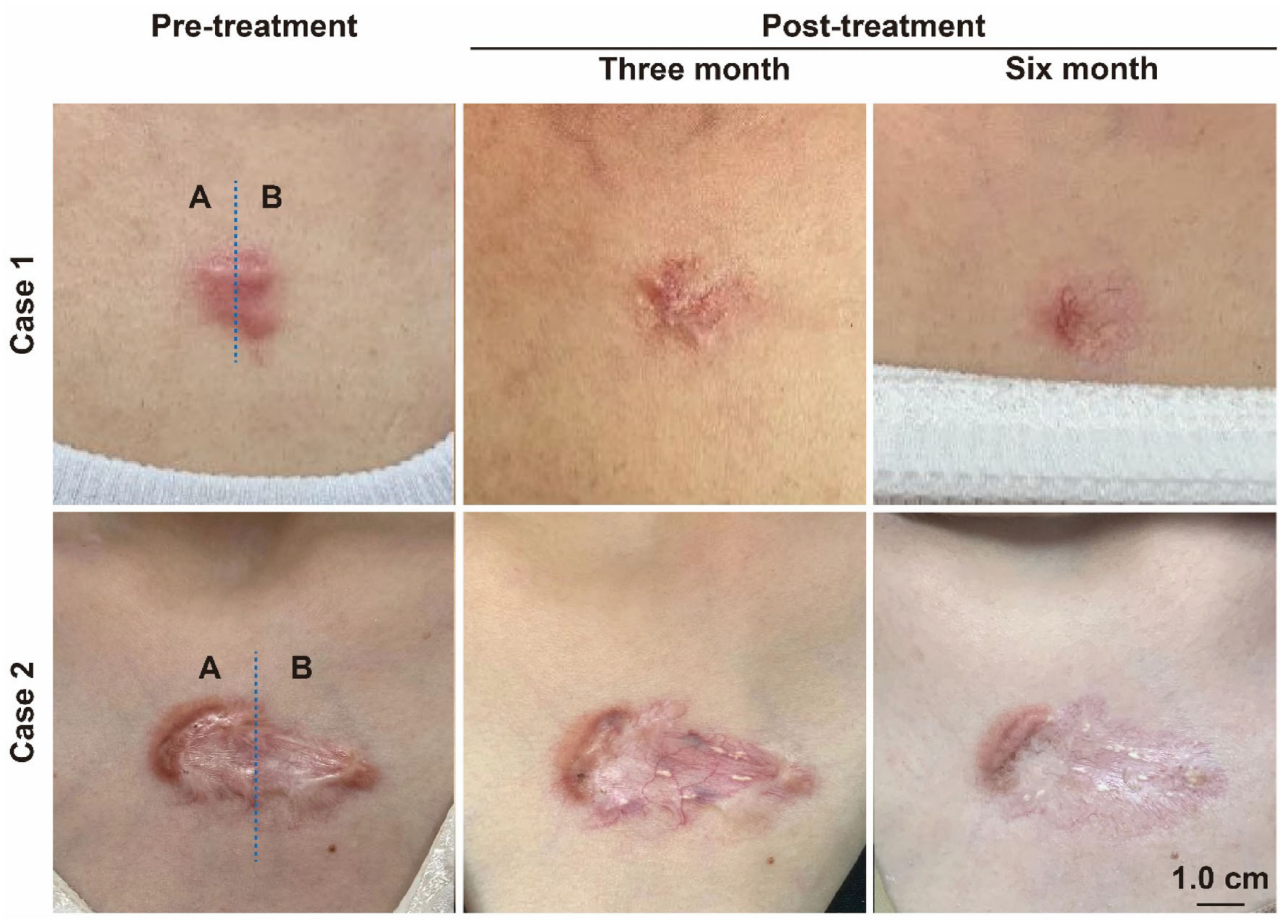
Case 1: a 33-year-old female patient with keloids in the anterior chest area; Case 2: a 25-year-old female patient with keloids in the anterior chest area; changes in the keloids before treatment, 3 months, and 6 months after receiving the new treatment (Zone B) versus the traditional medicine (Zone A). Scale bar, 1.0 cm

To further validate this treatment's therapeutic efficacy and exclude individual differences, this study used an auto control in which the same keloid in the same patient was divided into two, receiving the new treatment versus the traditional treatment protocol. We report two typical cases, as shown in Fig. 10: the improvement in keloid scars was more obvious in this treatment regimen than in the traditional treatment regimen at 3 and 6 months after the treatment, as seen under direct vision, and there were no relevant complications and related side effects, and no recurrence at 1-year follow-up, which is a remarkable efficacy. The present study achieved a more pronounced therapeutic effect than the traditional treatment, with a lower complication and recurrence rate than the traditional treatment and higher overall satisfaction; it is a feasible therapeutic option.

## Discussion

Using radionuclides combined with intralesional injections methods to treat keloids is simple, inexpensive, effective, and safe. However, complications such as rupture, pigment loss, and recurrence limit the popularization and application of this protocol. This method needs to be improved to reduce the complication rate and further improve its efficacy. We investigated the spatial conformational features and patterns of the significant components of keloid tissue, both in vivo and ex vivo, by single-cell sequencing analyses, ultrasonography, and molecular biology techniques.

To investigate the specific problem of keloid scarring, which is currently difficult to treat, single-cell sequencing analyses in this study revealed that genes and enriched pathways highly expressed in endothelial cells and mural cells, the two central cells of the vascular wall, play essential roles mainly in angiogenesis and in promoting collagen synthesis to promote keloid scar growth. Further experimental studies and analyses found that keloids have structural, vascular distribution, and closure differences at different levels. The first layer of the keloid has abundant blood vessels and other vascular systems. The blood vessels are open, and metabolism is vigorous, which provides fuel for its invasive growth; from the first layer to the third layer of the vascular system is gradually pressed by collagen fibers to close, the blood vessels and other vascular systems are reduced, and the lumens are gradually closed; the lumens of the third layer are less, and the metabolism is reduced. Blood vessels and other vascular systems play an important role in tissue growth and development, metabolism, and functional activities, which suggests a guiding role for the treatment of keloid scars.

Based on the findings of this study, the "sandwich" therapy of radionuclide combined with intralesional injections was rationally and skillfully designed, given the differences in the spatial structure of keloid scars and the distribution of blood vessels and other vascular systems in keloid scars. The first layer of keloid (Rich and open blood vessels, strong metabolism) 2–3 mm should be treated with radionuclide; the first layer of below 2 mm and the second layer (About 1/3–1/2; middle vessels and partially compressed lumen, middle metabolism) were given a second superficial intralesional injections of a mixture of TAC and 5-FU (5-FU : TAC = 2:1); the third layer (1/3 – 1/2; fewer vessels and completely compressed, weak metabolism) was given a third deep intralesional injections of a mixture of TAC and 5-FU (5-FU: TAC = 1:5). The main consideration is that both endothelial cells and fibroblasts are highly sensitive to radiotherapy, which can inhibit angiogenesis, which in turn prevents the formation of dysfunctional blood vessels, reduces inflammation, and destroys the nutrients of the tissue, and radiotherapy can inhibit the activity of fibroblasts, causing them to undergo denaturation, which reduces the synthesis and deposition of collagen, thus effectively treating keloids. However, ^90^Sr releases β-rays during the decay process and its invasion depth is shallow, only penetrating 2–3 mm below the skin surface. Therefore, in this study, the first layer below 2 mm was injected with a mixture of TAC and 5-FU, and the second layer and above were injected with a higher concentration of 5-FU in the shallow part of the second layer (5-FU: TAC = 2:1), while the deep part of the third layer was injected with a higher concentration of TAC (5-FU: TAC = 1:5); the main considerations were that the main effects of 5-FU could inhibit vascular regeneration, lead to vascular occlusion, and antimetabolic activity, whereas the main effects of TAC could inhibit the ability of fibroblasts to grow through apoptosis and inhibition of TGF-β1 expression, leading to degradation of collagen fibers. In addition, recent studies have shown that that the fibroblasts of keloid were differentiated from fascial stem cells, providing theoretical support for the treatment plan in this study, in which the third layer was injected deeper near the fascial layer to inhibit the differentiation of stem cells in the fascial layer and reduce fibroblasts, thereby improving the efficacy [9]. This treatment plan is based on the differences in the spatial structure of keloid components to adjust the injection scheme and drug ratios to achieve a more targeted and precise treatment, which can achieve satisfactory therapeutic effects; TAC and 5-FU have a synergistic effect when used in combination, which can improve the effectiveness and safety of keloid treatment [10].

In addition, the "sandwich" therapy, which is rationally designed at the keloid structure level, only requires radionuclide to treat superficial 2 - 3 mm keloid, reduce recurrence, and maintain stability, so there are no need to worry about the complications of shallow radionuclide invasion and no need to wait for the keloid to atrophy after intralesional injections before radiotherapy. In the present study, radiotherapy was started the next day after intralesional injections, which shortened the treatment period, with higher practicability, greater patient compliance, and significant overall efficacy, as well as avoiding the problem of insufficient penetration.

In conclusion, the "sandwich" therapy designed in this study based on the structural hierarchy of keloids and their vascular distribution is a highly targeted and precise therapeutic solution for efficiently treating keloids. The minimally invasive treatment strategy consists of only three intralesional injections of TAC + 5-Fu and radionuclide. The treatment program largely reduces the pain, inconvenience, and complications associated with prolonged and repeated injections of TAC or 5-Fu, and improves patient compliance, resulting in high patient satisfaction. In contrast with other studies focusing on treating hyperplastic scars or keloids after their formation, the present study reveals the structural hierarchy of keloids, and based on this, a rationally designed and highly targeted "sandwich" therapy is safe and effective for treating scars. This implies that the “sandwich” therapy of radionuclide combined with intralesional injections based on differences in the spatial structure of keloid scars is an improved keloid treatment worthy of clinical promotion.

## Conclusion

We found that keloid scars have a typical structural hierarchical difference, from the first to the third layer of blood vessels gradually reducing, and the lumen gradually from open to closed. Based on this difference in the spatial structure of keloid, the "sandwich" therapy of radionuclide combined with intralesional injections is reasonably designed, which is highly targeted, with a short course of treatment and reasonable use of the synergistic effect of combined therapy, with remarkable efficacy, high patient compliance, and high satisfaction. At the same time, we believe that the highly targeted treatment program based on the differences in the spatial structure of keloid scars has a very strong targeted treatment with high spatial and temporal accuracy and minimal invasiveness and is a promising method for the treatment of keloid scars.
